# *Monotheca buxifolia* Driven Synthesis of Zinc Oxide Nano Material Its Characterization and Biomedical Applications

**DOI:** 10.3390/mi13050668

**Published:** 2022-04-24

**Authors:** Muhammad Ishaq Khan, Sumaira Shah, Shah Faisal, Safia Gul, Shahzar Khan, Abdullah, Sajjad Ali Shah, Wajid Ali Shah

**Affiliations:** 1 Department of Botany, Bacha Khan University, Charsadda 24460, Pakistan; sunehra23@gmail.com; 2 Department of Health and Biological Sciences, Abasyn University, Peshawar 25000, Pakistan; shahfaisal11495@gmail.com; 3 Institute of Biotechnology and Microbiology, Bacha Khan University, Charsadda 24460, Pakistan; sajjadbiotec@gmail.com; 4 University College for Women, Abdul Wali Khan University, Mardan 23200, Pakistan; safiagul@awkum.edu.pk; 5 Department of Microbiology, Abdul Wali Khan University, Mardan 23200, Pakistan; shahzarkhankmu@gmail.com (S.K.); abdul.9353chd@gmail.com (A.); 6 Department of Agriculture, Bacha Khan University, Charsadda 24460, Pakistan; wshah75@bkuc.edu.pk

**Keywords:** *Monotheca buxifolia*, zinc oxide nanoparticles, anti-fungal, anti-Alzheimer’s, anti-diabetic

## Abstract

The current study demonstrates a sophisticated and environmentally friendly synthesis of zinc oxide nanoparticles (ZnO-NPs) for a range of biological and environmental applications using *Monotheca buxifolia* as a bio-source. At the nanometer scale, a simple aqueous extract from *Monotheca buxifolia* was used to convert Zn into stable elemental zinc (Zn^0^). With an average size of 45.8 nm and a spherical shape, the NPs were stable and pure. The nanoparticles studied here were tested in vitro for bactericide, fungicide, biocompatibility, leishmaniasis, anti-diabetic effect, antioxidant effect, and anti-Alzheimer’s effect. According to our results, *Monotheca buxifolia* mediated ZnO-NPs are highly effective against spore-forming fungal strains and MDR bacterial strains. All examined bacterial isolates of UTI (urinary tract infection) were resistant to non-coated antibiotics; however, adding 1% of the produced ZnO-NPs to the treatments increased their bactericidal activity significantly. The NPs also showed dose-dependent cytotoxicity against Leishmania tropica parasites, with an LC50 of 248 μg/mL for promastigote parasites and 251 μg/mL for amastigote parasites. In addition, a significant inhibition of α-glucosidase, α-amylase, butyrylcholinesterase (BChE), and acetylcholinesterase (AChE) was discovered, indicating anti-Alzheimer’s and anti-diabetic effects. The biocompatibility of the particles with human red blood cells was also observed. Due to their environmentally friendly production, biological safety, and exceptional physicochemical properties, ZnO-NPs could be used as a new competitor for several biological and environmental applications.

## 1. Introduction

The development of green, environmentally friendly, and reliable manufacturing processes for nanomaterials has been a growing trend in nanoscience and technology over the past decade [1]. Zinc nanoparticles have aroused the interest of the scientific community due to their many uses in plasmonics, cosmetics, catalysts in the biomedical field, pure water technologies, data storage, optoelectronics, sensors, and textiles [2,3]. Zinc nanoparticles have excellent bio-optical and physicochemical properties, making them among the most widely used nanoscale materials for wound healing, dental applications, catheter modification, cancer therapy, drug delivery, and anti-viral, anti-inflammatory, anti-angiogenesis, and antimicrobial agents, to name just a few applications [4,5]. Both bottom-up and top-down techniques are often used in the manufacture of ZnO-NPs. Chemical processes such as solvothermal, co-precipitation, sonochemistry, pyrolysis, and green approaches are examples of bottom-up processes, while pulsed laser ablation, spray pyrolysis, and ball milling are examples of top-down processes [6]. Traditional approaches such as chemical treatments and top-down tactics are inefficient, polluting, and time-consuming. Scientists want to synthesize ZnO-NPs from bacteria, fungi, and plants in order to circumvent these limitations [7].

Plant-mediated synthetic technologies with lower environmental impact are being developed. In recent years, nanomaterials scientists have given priority to developing eco-friendly manufacturing processes for nanoscale materials. The synthesis of green nanoparticles, especially using plant extracts, is a relatively new approach in environmentally friendly chemistry that promises to be simple, non-toxic and inexpensive [8]. Nanotechnology has improved people’s lives by addressing a variety of challenges and playing a key role in the treatment of a wide variety of diseases [9]. Zinc oxide nanoparticles are useful in a variety of new applications, including catalysis, petrochemical extraction, adsorption, and electronics, due to their superior crystal structure and ionic properties [10]. There are several options, but the green approach is the best. The physiomorphic properties of ZnO nanoparticles, such as size, shape, and crystallinity, are influenced by the reaction time and reaction conditions [11]. Due to the limitations of non-green synthesis methods mentioned above, nanomaterials scientists are now using the immense potential of medicinal plants as a reliable basis for the production of ZnO-NPs as a viable alternative to conventional processes [12].

*Monotheca buxifolia* belongs to the genus *Monotheca* and the family *Sapotaceae*. *M. buxifolia* is an extremely important tree species in Pakistan and continues to dominate areas of the country’s forests, particularly in the Dir district. It is also found in the highlands of Northern Oman, Southeast Saudi Arabia, and Afghanistan [13]. Because of its thorny character, *M. buxifolia* is commonly used for smaller lumber, roofing material, fuel, and fodder and as a barrier around agricultural regions. This species also produces fruits known in the area as gurguri, which are a source of income for the locals [14]. *M. buxifolia* leaf is used in traditional medicine as an antipyretic, laxative, vermicide, hematin, laxative, and treatment for gastrointestinal disorders [14,15]. For the first time, we have examined *M. buxifolia* synthesized ZnO-NPs alone and as coating with antibiotics in our research against urinary tract infection isolates. In addition, there is little information in the literature on the anti-Alzheimer’s properties of green fabricated ZnO-NPs. With these shortcomings in mind, the well-characterized NPs were tested for a variety of biological applications.

## 2. Materials and Methods

### 2.1. Monotheca buxifolia Extract Preparation

The herb used in this study was collected in the Charsadda District. The plant was recognized as *Monotheca buxifolia*, which was later validated at Pakistan’s Bacha Khan University, Department of Botany. Fresh and fine plant leaves were cut into little pieces with a sterile medical blade and then thoroughly cleaned with distilled water to remove any dust or impurities before drying in the shade. For aqueous extraction, the well-dried leaves were ground in a Willy mill and stored at 25 °C. To generate an aqueous solution, fine powder up to 30 g of plant material was added to flasks (500 mL) containing distilled water (200 mL). Flasks were sonicated for 10 min and kept at 37 °C for two days in a shaking incubator at 200 rpm. The produced extract was filtered twice with nylon cloth and three times with Whattman filter paper to eliminate any remaining particles. The size of the Whatman filter was 20 μm. Following that, the fresh filtrate was recycled for future use.

### 2.2. Biosynthesis of Zinc Oxide Nanoparticles (ZnO-NPs)

An extract of the *Monotheca buxifolia* leaf was used to synthesize ZnO nanoparticles. Zinc nitrate was added in the following ratio: 1:1 (pH: 5.7). The reagent was properly stirred using a magnetic stirrer at 60 °C for two hours. The change in color showed that nanoparticles had been synthesized. Centrifugation at 10,000 rpm for 17 min was used to separate the color-changed solution. The supernatant was thrown away, and the pellets were saved in a separate container for later use. The pellets were recovered after centrifugation and cleansed with ethanol before being calcinated for two hours at 500 °C to remove contaminants. The nanoparticles were kept at 4 °C until they were needed again. 

### 2.3. Characterization of Biosynthesized ZnO-NPs

Advanced methods such as X-ray diffraction (XRD), energy-dispersive X-ray, scanning electron microscopy, UV-visible spectroscopy, and Fourier transform infrared spectroscopy were used to assess the physicochemical and morphological features of produced ZnO-NPs [15]. UV spectroscopy uses the conventional and typical range of 200 to 700 nm to monitor the biosynthetic and environmentally friendly interaction between plant extract and metallic salt. The crystal nature of biosynthesized NPs was determined using X-ray diffraction technique. Panalytical’s X’Pert X-ray diffractometer was used to produce the XRD peaks at CuK (=1.54056) (step-time of 0.55 sec and scanning step size of 0.03°/s). The average grain size of Ni-NPs was calculated using Scherer’s equation.

D=kλ/βCosθ

ZnO-NPs were studied using FTIR spectroscopy in the 400–4000 cm^−1^ spectrum region to identify and assess related functional groups involved in their biogenesis. SEM was used to measure the physical dimensions and morphological properties of biosynthesized ZnO-NPs (JSM-5910, Tokyo, Japan). The biosynthesized ZnO-NPs were subjected to energy-dispersive X-ray spectroscopy to determine their elemental composition.

### 2.4. Antibacterial Assay

#### 2.4.1. Collection of Bacteria

*Pseudomonas aeruginosa*, *Staphylococcus aureus*, *Klebsiella pneumonia* and *E. coli* were among the four multidrug-resistant bacteria found in urinary tract infection patients. These isolates were collected from the HMC (Hayat Abad Medical Complex in Peshawar) and were previously identified by biochemical testing and a molecular technique (16s rRNA) at the hospital.

#### 2.4.2. Preparation of Antibiotic Discs Coated with ZnO-NPs

To create the powdered ZnO-NPs stock solution, 15 mg of ZnO-NPs residual was dissolved in 1 mL of distilled water. After thorough mixing, 5 μL of the suspension was transferred to antibiotic discs within petri plates, which were then dried in an oven at 80 °C for 15 min. Each antibiotic was coated using the same method.

#### 2.4.3. Agar Well Diffusion Assay for ZnO-NPs

In the bacterially lawned nutrient agar (NA) medium, 8 mm diameter wells were punched according to [16]. A 100 µL suspension of ZnO-NPs was used to fill the wells. The petri plates were then incubated for 24 h at 37 °C. The potential of ZnO-NPs against the MDR bacterial strains studied was determined early after incubation by measuring inhibitory zones in mm.

#### 2.4.4. Disc Diffusion Assay for ZnO-NPs Coated and Non-Coated Antibiotics

The conventional Kirby Bauer disc technique was used to assess the efficacy of coated and non-coated antibiotic discs against MDR bacterial strains. After making nutrient agar, the NA medium and petri plates were autoclaved according to standard operating procedures. The medium was sterilized and then refrigerated to 50 °C before being transferred to sterile petri plates in the biosafety cabinet. After the culture had hardened, a bacterial lawn was created on nutrient agar plates, followed by the application of both non-coated and coated ZnO-NPs antibiotic discs, and incubation at 37 °C for 24 h. After incubation, the effectiveness of non-coated and coated antibiotics against test MDR bacterial strains was measured in mm. 

### 2.5. Antifungal Assay

ZnO-NPs were tested against five spore-forming fungus including *Fusarium solani* (FCBP 434), *Aspergillus fumigatus* (FCBP 66), *Mucor species* (FCBP 300), *Aspergillus niger* (ATCC 1015), and *Aspergillus flavus* (ATCC 1015) [16,17]. A 20 µL solution (0.02% *v*/*v*) was used to make spore suspension from stock cultures for each fungal strain. A 100 µL aliquot of the suspension was swabbed well and put on several petri plates with sterilized SDA medium. Following that, testing samples of 10 µL were inserted into each well inside the solidified medium under very sterile conditions. For zone of inhibition investigations, DMSO and Clotrimazole were used as negative and positive controls, respectively. After 48 h, inhibition zone were measured using a Vernier caliper. 

### 2.6. Anti-Leishmanial Assay

The leishmanicidal potential of ZnO-NPs against both promastigote and amastigote were investigated using a well-established method [18]. The *Leishmania* KWH23 strain was grown and incubated in MI99 media supplemented with 10% FBS. In total, 20 µL of NPs and 180 µL of culture solution were placed in each well of a 96-well plate. The mixture was incubated for 72 h at 25 °C. Amphotericin was used as positive control, and DMSO was used as negative control. After incubation, each well was filled with 20 µL of MTT solution (4 mg/mL in dH_2_O), and the culture plate was re-incubated at 25 °C for another 4 h. A 96-well microplate reader was used to measure the absorbance at 540 nm. The following formula was used to compute the percent inhibition:

$$\%\;\mathrm{Inhibition}=\left[1-\left\{\frac{\mathrm{Absorbance}\;\mathrm{of}\;\mathrm{sample}}{\mathrm{Absorbance}\;\mathrm{of}\;\mathrm{control}}\right\}\right]\times 100$$

### 2.7. Anti-Alzheimer’s Assay

The capacity of ZnO-NPs to inhibit acetylcholinesterase (AChE) and butyrylcholinesterase (BChE) was checked at different concentrations ranging from 62 to 1000 µg/mL [19]. Phosphate-buffered saline, 5,5-dithiobisnitrobenzoic acid, acetylcholine iodide, and butyrylcholine iodide were used to make a substrate solution. The pristine reaction mixture was employed as a positive control, while galanthamine hydrobromide (5 µg/0.5 mL methanol) was used as a negative control. Finally, the absorbance of the samples at 412 nm was determined using a UV-VIS spectrophotometer. The inhibitory activities of enzymes were calculated using the following equation:

$$\begin{array}{c} \%\;\mathrm{Enzyme}\;\mathrm{activity}=\left(\frac{V}{V\;\max}\right)\times 100\\ \%\;\mathrm{Enzyme}\;\mathrm{inhibition}\;=100-\%\;\mathrm{Enzyme}\;\mathrm{activity} \end{array}$$

### 2.8. Protein Kinase Inhibition Assay

To evaluate the protein kinase inhibition potentials of biogenic ZnO-NPs, previously established technique was used with minimal adjustments [20]. In this work, Streptomyces 85E strain was cultured using sterile ISP4 media. Then, wells were produced in media, and 100 µL of NPs were added to each well. As positive control, surfactin was employed, whereas for negative control, DMSO was used. The plates were then incubated for two days at 28 °C. After two days a clean and bald zone around wells was measured by Vernier caliper. 

### 2.9. Anti-Diabetic Assay

The anti-diabetic efficacy of ZnO-NPs was evaluated using α-amylase and α-glucosidase inhibition tests [21].

#### 2.9.1. α-Amylase Inhibition Assay

The potential of ZnO-NPs was assessed using α-amylase test via a well-established technique with only minor changes [22]. A 96-well microplate was used for this experiment. Each well in the test was filled with α-amylase (25 µL), phosphate buffer (15 µL), starch (40 µL), and ZnO-NPs sample (10 µL) and then incubated for 30 min at 50 °C. Next, 20 µL of 1M HCl and 90 µL of iodine solution were loaded into each well. In this experiment, Acarbose was employed as a positive control, and DMSO was used as a negative control. The absorbance of the test samples was recorded at 540 nm. Using the following formula, the inhibition was determined as a percentage:

$$\%\;\mathrm{Enzyme}\;\mathrm{inhibition}=\left(\frac{\mathrm{Abs}\;\mathrm{Sample}-\mathrm{Abs}\;\mathrm{negative}\;\mathrm{control}}{\mathrm{Abs}\;\mathrm{blank}-\mathrm{Abs}\;\mathrm{negative}\;\mathrm{control}}\right)\times 100$$

#### 2.9.2. α-Glucosidase Inhibition Assay

The α-glucosidase inhibition potentials of ZnO-NPs were evaluated using a modified protocol [23,24]. To dissolve α-glucosidase, 50 mL of phosphate buffer (pH 6.8) and 100 mg of bovine serum albumin were poured simultaneously. After 5 min, 490 μL of phosphate buffer (pH 6.8) and 250 μL of p-nitrophenyl-D-glucopyranoside (5mM) were mixed and then kept at 37 °C. After that, ZnO-NPs were treated with 250 μL α-glucosidase for 15 min at 37 °C. After stopping the reaction with a 2 mL Na_2_CO_3_ (200 mM) solution, absorptions were measured at 400 nm. The experiment was repeated three times, and in this experiment, acarbose was used as a positive control.

(1) $$\%\;\mathrm{Enzyme}\;\mathrm{inhibition}=\left(\frac{\mathrm{Abs}\;\mathrm{Sample}-\mathrm{Abs}\;\mathrm{negative}\;\mathrm{control}}{\mathrm{Abs}\;\mathrm{blank}-\mathrm{Abs}\;\mathrm{negative}\;\mathrm{control}}\right)\times 100$$

### 2.10. Biocompatibility Studies

Fresh human red blood cells (hRBCs) were used to test the biocompatibility of ZnO-NPs [25]. An amount of 1 mL of blood was collected from a healthy person and deposited in an EDTA tube after obtaining their permission. Blood samples were centrifuged for 7 min at 12,000 rpm to remove red blood cells. The particle was recovered after three PBS washes, and the supernatant was discarded. To generate a PBS-erythrocyte suspension, 200 μL of erythrocytes were combined with 9.8 μL of PBS (pH: 7.2). Erythrocyte suspension and various concentrations of biosynthesized NPs were mixed in Eppendorf tubes and incubated for 1 h at 35 °Celsius. After centrifuging the mixture at 1000 rpm for five minutes, the supernatant was transferred to a 96-well plate, and hemoglobin release absorption spectra at 540nm were recorded. As positive control of 0.5 percent Triton X-100 was utilized, whereas DMSO was used as a negative control. The following formula was used to determine the fraction of hemolysis:

$$\left(\%\right)\;\mathrm{Haemolysis}\;=\left(\frac{\mathrm{sample}\;\mathrm{Ab}-\mathrm{negative}\;\mathrm{control}\;\mathrm{Ab}}{\mathrm{Positive}\;\mathrm{control}\;\mathrm{Ab}-\mathrm{Negative}\;\mathrm{control}\;\mathrm{Ab}}\right)\times 100$$

Here, Ab stands for the absorbance of the samples as recorded.

## 3. Results

### 3.1. Synthesis of Zinc Oxide Nanoparticles Using Monotheca buxifolia Extract

**Figure 1:**
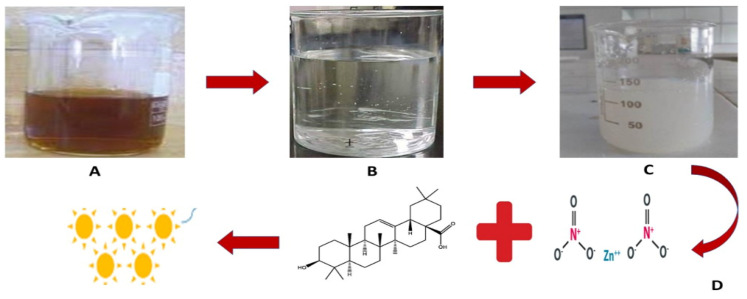
(**A**) Aqueous extract of *Monotheca buxifolia.* (**B**) Salt solution of zinc nitrate. (**C**) reduction (**D**) Mechanistic approach for the synthesis of ZnO-NPs through plant active compounds.

After isolating and confirming the *Monotheca buxifolia* leaves at the Department of Botany, Bacha Khan University, Pakistan, they were washed three times and air dried in the shade before being ground to a fine powder. A total of 10 g of dried leaves powder was mixed with 100 mL of deionized water and reacted at 60 °C for 15 min. After reaction, the extract was filtered three times using nylon cloth and Whatman filter paper (20 μm), and any residue was removed by centrifugation at 12,000 rpm. The pellets were discarded, and the supernatant was collected and stored in separate tubes at 7 °C for use in the experiment. In total, 6.0 g of zinc nitrate were added to 100 mL of the extract on a magnetic stirrer, and the mixture was kept at 60 °C for 2 h. The color change from green to white, as can be seen in Figure 1, is an early sign of the formation of zinc nanoparticles. After a sufficient reduction step, the solution was centrifuged at 12,000 rpm for 20 min to obtain pure nanoparticles. The pellets were rinsed three times with distilled water and dried in an oven at 80 °C. The dried nanoparticles were stored at 4 °C for further use and characterization.

### 3.2. UV-Analysis

**Figure 2:**
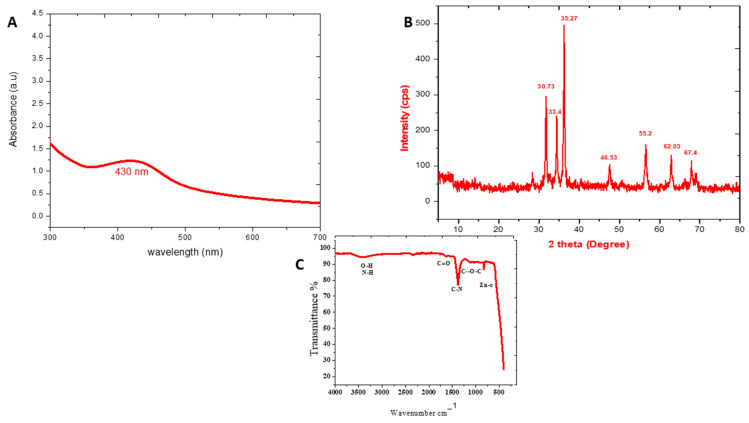
(**A**) UV visible spectrum. (**B**) Typical XRD pattern. (**C**) Typical FTIR spectra of *Monotheca buxifolia* synthesized ZnO-NPs.

The reaction mixture’s initial color change from green to grey-white suggests that bioactive chemicals in the *Monotheca buxifolia* aqueous extract reduced Zn ions to Zn^0^. After the color change, the reaction mixture was allowed to cool for 40 min to allow for proper reduction before being tested for absorbance using a spectrophotometer at a specified wavelength (Shimadzu UV-1800). Figure 2A shows that the zinc oxide nanoparticles absorbed some of the characteristic wavelength, resulting in a surface plasmon resonance peak at 430 nm. 

### 3.3. XRD Analysis

The crystalline structure of zinc oxide nanoparticles was investigated using an X-ray diffractive analysis (JDX-3532, JEOL, Tokyo, Japan). Numerous XRD reflection planes at 2 Theta or diffraction angles such as 30.73°, 33.4°, 35.27°, 46.53°, 55.2°, 62.03°, and 67.4° were observed, as shown in Figure 2B. After basic calculations, these peaks indicate the face-centered cubic crystal structure of ZnO-NPs. The average crystal size of ZnO-NPs is 45.32 nm according to Scherer’s equation (see below), D ¼ kk = bCosh.

### 3.4. FTIR Analysis

**Figure 3:**
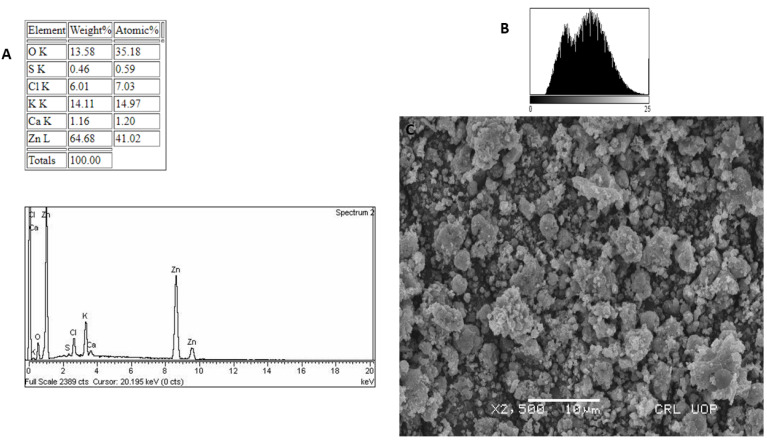
(**A**) EDX spectrograph. (**B**) Histogram. (**C**) SEM micrograph of Monotheca buxifolia synthesized ZnO-NPs.

The surface chemistry of ZnO-NPs and more specifically engaged functional groups of plant extract were determined using the FTIR method. Figure 2C shows an example of this. The large peak seen at 3408 cm^−1^ in bio-assisted ZnO-NPs may be attributed to protein NH (amide A) or OH stretching. C=O stretching vibrations of the protein amide-I bonds provide a large peak at about 1640 cm^−1^. The stretching of the C–N bond of the amino acid was ascribed to the absorption bands at 1394 cm^−1^ in Figure 3. The polysaccharide carbohydrate C–O–C ether bond displays vibration at around 1038 cm^−1^. At 517 cm^−1^, the Te absorption peak associated with the Zn-O stretching band is clearly visible, confirming the creation of ZnO-NPs. 

### 3.5. EDX and SEM Analysis

A significant EDX spectrum for zinc was detected, as shown in Figure 3A, demonstrating the formation of zinc oxide nanoparticles. Extra peaks may be seen of chlorine, carbon, oxygen, phosphorus, sulphur, potassium, calcium, sodium, and magnesium. The additional peaks were created by the bioactive chemicals in *Monotheca buxifolia* that surrounded the Zn ions during nanoparticle formation. The physical dimensions and diameters of the zinc nanoparticles were determined using scanning electron microscopy. SEM (JSM5910) was used in this investigation. Despite the white spots seen in the SEM micrograph (Figure 3B), some particles are homogenous, while others are polydispersed, with an average size of 45.8 nm confirmed by ImageJ and Nano Measurer analysis.

### 3.6. Antibacterial Activity of ZnO-NPs

In this work, pure ZnO-NPs and antibiotic discs coated with ZnO-NPs were tested against UTI isolates such as *K. pneumoniae*, *S. aureus*, *E. coli*, and *P. aeruginosa*. Pure ZnO-NPs had the greatest activity against ZnO-NPs against *S. aureus* (18 ± 2.74), followed by *K. pneumoniae* (16 ± 2.78), *E. coli* (14 ± 2.63), and *P. aureginosa* (13 ± 2.43). All strains showed resistance to non-coated ciprofloxacin, imipenem, vancomycin, clavulanic acid, and amoxicillin. In our investigation the antibiotic disc coated with ZnO-NPs showed increased antibacterial potential against bacterial strains. For instance, ciprofloxacin had a 29% increase in potency against *K. pneumoniae*, imipenem increased up to 25.2%, clavulanic acid had a 22.6% activity, and Vancomycin had an 18.0% activity. Ciprofloxacin’s bactericidal activity against *S. aureus* was enhanced to 29.5%, vancomycin’s to 26.9%, imipinem’s to 22.5%, and amoxicillin-clavulanic acid to 17.3%. In *P. aureginosa*, ciprofloxacin sensitivity increased to 27.9%, imipinem sensitivity to 26.6%, amoxicillin-clavulanic acid sensitivity to 21.4%, and Vancomycin sensitivity to 13.8%. As indicated in Table 1, the gram-negative MDR strain of *E. coli* showed a significant increase in sensitivity to ZnO-NPs coated medications. 

**Figure 4:**
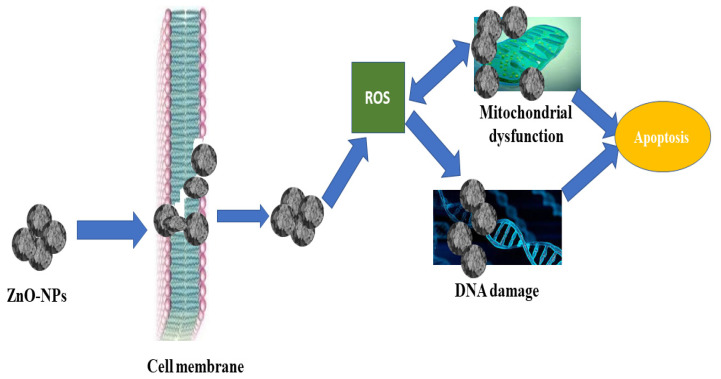
Mechanistic Approach of ZnO-NPs for Cytotoxicity.

Zinc oxide nanoparticles have been proposed as means of action against bacteria and fungi based on the formation of reactive oxygen species (ROS) as a result of light, the breakdown of metal oxide nanoparticles, and the electrostatic correspondence of nanoparticles with microbe cell walls. The antibacterial action of ZnO-NPs might be due to their aggregation predominantly on the outside of the bacterial membrane. ZnO-NPs produce reactive oxygen species (ROS), which bind to the bacterial membrane and cause permeability and cell death. The release of Zn4+ damages bacteria’s mitochondria and DNA, and it is capable of blocking important bacterial enzymes, resulting in cell death, as seen in Figure 4.

### 3.7. Antifungal Activity

**Figure 5:**
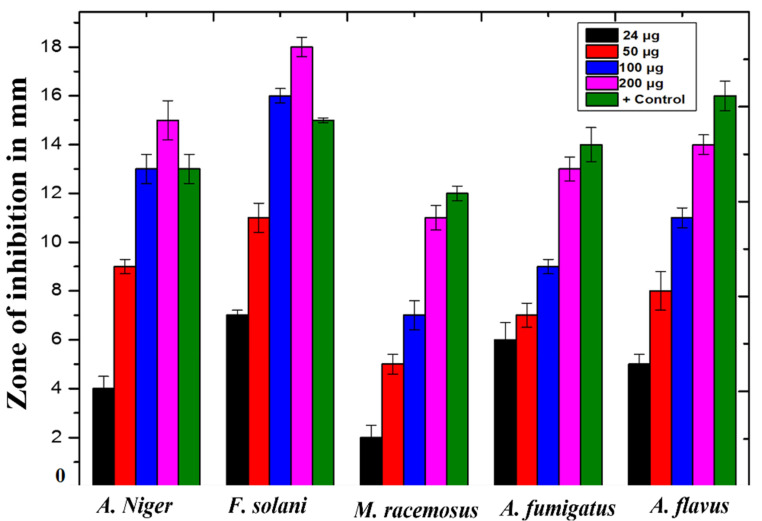
Antifungal activity of *Monotheca buxifolia* synthesized ZnO-NPs.

Using well diffusion methods, the antifungal potential of ZnO-NPs was studied. The outcomes are depicted in Figure 5. In this study, ZnO-NPs were evaluated against five filamentous fungal strains at stock concentrations ranging from 25 µg/well to 200 µg/well. As a positive control, amphotericin B was used, while DMSO was used as a negative control. The antifungal activity of all the samples was dose-dependent. Values of 14.5 ± 2.5 mm, 18.2 ± 1.5 mm, and 13.8 ± 2.17 mm were found for *A. nigr*, *F. solani*, and *A. flavus* at 200 µg/well, respectively. *M. racemosus* had the lowest inhibition zone of 11.52.3 mm.

### 3.8. Anti-Leishmanial Activity

**Figure 6:**
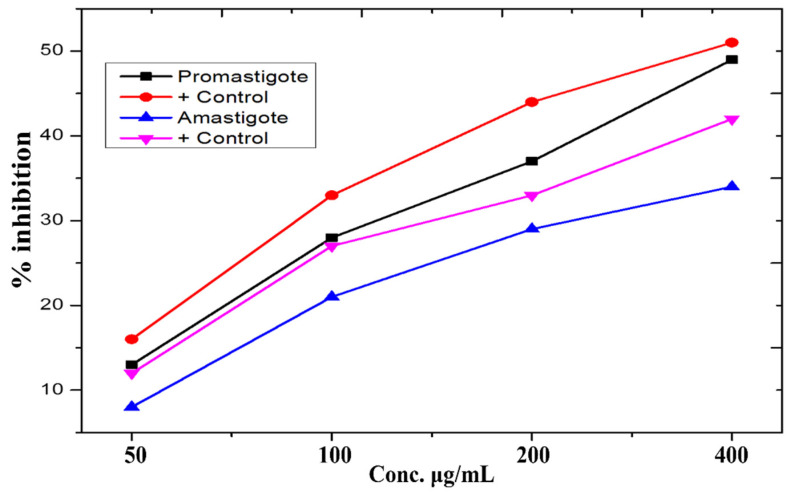
% Mortality of amastigote and promastigote parasites potential of ZnO-NPs.

In this work, ZnO-NPs formulations ranging from 50 to 400 μg/mL were tested against promastigote and amastigote cultures of *L. tropica* using the MTT assay. At 400 μg/mL, cytotoxicity was dose-dependent, with substantial mortality rates of 49 ± 0.67% and 34 ± 0.93% for the promastigote and amastigotes forms of the parasite, respectively, as shown in the Figure 6. The anti-leishmanial potency of ZnO-NPs in concentrations ranging from 50 μg/mL to 400 μg/mL was tested. Metal oxides, according to recent research, offer great potential as anti-leishmanial agents. We can deduce from our findings that leishmania cytotoxicity is dosage dependent, with amastigotes being more susceptible to infection than promastigotes.

### 3.9. In Vitro Anti-Alzheimer’s Activity

**Figure 7:**
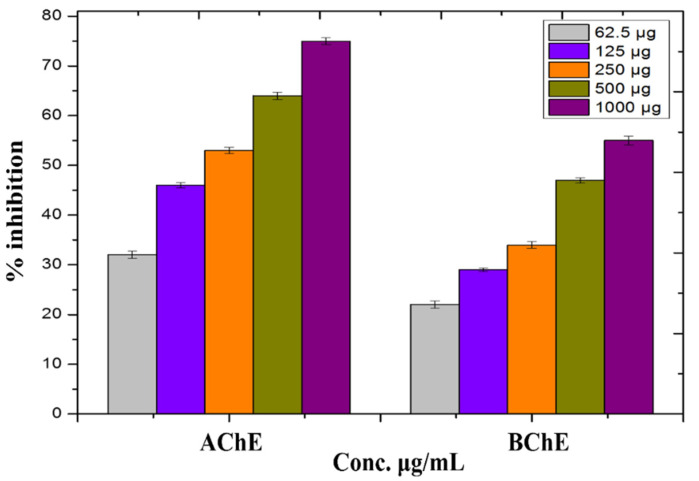
In-vitro cholinesterase potential of ZnO-NPs.

In this experiment, the capacity of ZnO-NPs to inhibit acetylcholinesterase (AChE) and butyrylcholinesterase (BChE) was checked at different concentrations ranging from 62 to 1000 μg/mL (Figure 7). Both enzymes’ inhibitory responses were found to be dosage-dependent. At 1000 μg/mL, the NPs inhibited AChE by 55.42 ± 0.21% and BChE by 75.13 ± 0.69%, respectively. At 62.5 μg/mL, AChE exhibited a 22.15 ± 0.57% inhibition reaction, whereas BChE showed a 32.23 ± 0.17 percent inhibition reaction. The particles were particularly potent against both cholinesterase enzymes.

### 3.10. Protein Kinase Inhibition Potential of Biosynthesized ZnO-NPs

**Figure 8:**
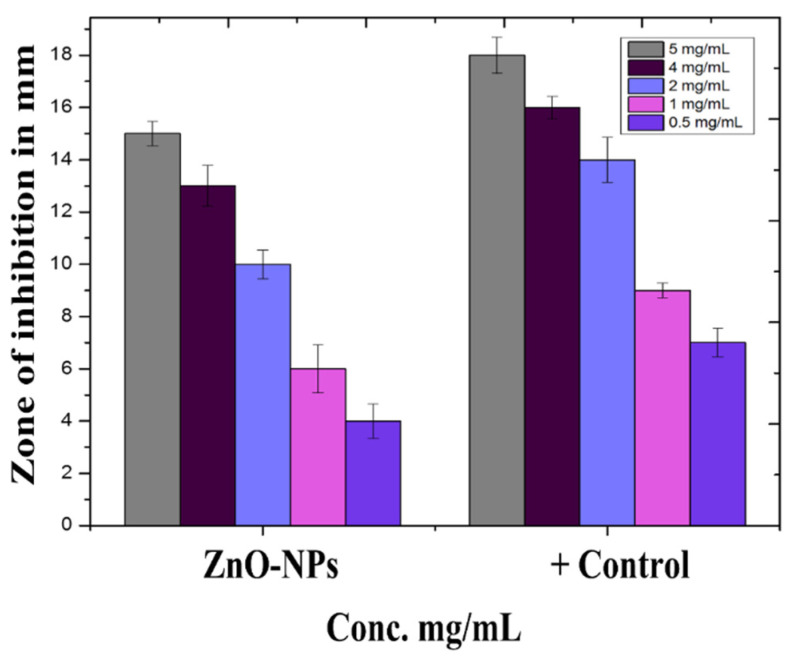
Protein Kinase Inhibition (in mm) by ZnO-NPs.

In *Streptomyces* 85E strain, ZnO-NPs at various doses ranging from 0.5 mg/mL to 5 mg/mL were tested for PK inhibition. For NPs formulations, dose-dependent inhibitory effect was established. The highest zone of inhibition was determined to be 15.2 ± 0.13 at 5 mg/mL, while the lowest zone of inhibition was calculated to be 4.3 ± 2.1 at 0.5 mg/mL, which is shown in Figure 8.

### 3.11. Anti-Diabetic Activity

**Figure 9:**
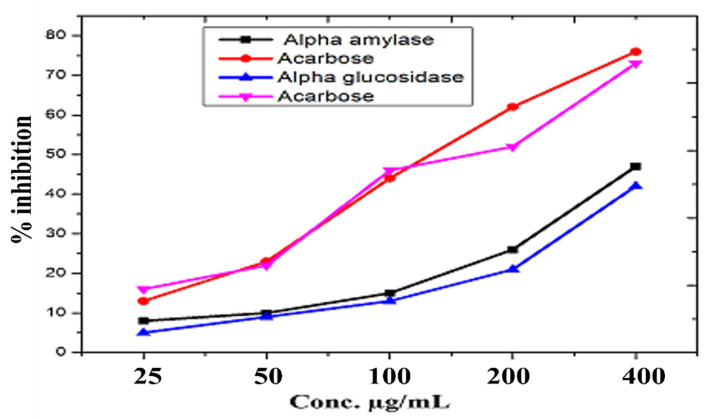
α-amylase and α-glucosidase inhibitory potential of ZnO-NPs.

As indicated in Figure 9, different concentrations of NPs ranging from 400 μg/mL to 25 μg/mL were tested for α-amylase and α-glucosidase inhibition. NPs significantly inhibit α-amylase and α-glucosidase. At the highest concentration of 400 μg/mL, maximum inhibition of 47.14 ± 0.24% for α-amylase and 42.69 ± 1.92% for α-glucosidase was found, with IC50s of 412 μg/mL and 457 μg/mL, respectively.

### 3.12. In Vitro Biocompatibility Study

Nanoparticles were evaluated for hemolysis against hRBCs to see whether they had any toxicological potential. Freshly produced hRBCs were co-incubated with ZnO-NP formulations (50 μg/mL to 400 μg/mL). Table 2 summarizes the findings of the assay. As indicated in the table, ZnO-NPs were hemocompatible, with no evident hemolytic activity even at the greatest stock concentration. No hemolytic activity was detected even at the highest concentration of 400 μg/mL, showing that the particles were hemocompatible. As a result, our findings support the particles’ biosafety and suggest therapeutic applications for ZnO-NPs.

## 4. Discussion

Because of its simplicity of use, nontoxicity, speed, and cost efficiency, as well as its potential for large-scale manufacturing, the green nanoparticle synthesis approach has piqued the scientific community’s attention in recent years. We used a green synthesis approach to make ZnO-NPs from a *Monotheca buxifolia* extract, and we tested the NPs for the first time, both as coating with antibiotics and alone. Urinary tract infection (UTI) isolates have never been found on the particles. Furthermore, there is no evidence in the literature to support the potential anti-Alzheimer’s effects of green-produced ZnO-NPs. The well-characterized NPs were put to the test in a variety of biological applications, with flaws factored in. In addition, ZnO-NPs were characterized using a variety of analytical techniques, including UV-visible spectroscopy, FTIR, SEM, EDX, and XRD. According to UV-visible spectroscopy, the sample absorbed energy at 430 nm, which is a typical peak value for ZnO-NPs. The results were validated by X-ray spectroscopy [26]. Aside from that, an absorption peak at 430 nm with no other peak demonstrated the nanoparticles’ exceptional purity. Many investigations have revealed a significant absorption peak of ZnO-NPs below 450 nm wavelength, which was related to the sample’s red shift at 500 and 700 degrees Celsius. They have also shown that in materials transitions, when an electron obtains energy, it transitions from a lower to a higher energy level [27]. The vibrations of alkanes, phenol, alcohols, aromatics, alkenes, alkyl halides, and aliphatic amines were revealed by FTIR analysis of zinc nanoparticles. Reference [28] also reported on similar results. Furthermore, –C=O–, C–O–C, and C–O stretching vibrations were shown to generate maxima in carboxylic acid, polysaccharide, and amino acid, respectively [29]. The same results were found. The created ZnO-NPs had particle sizes in the range of 45.8. nm, as estimated by Nano Measurer and ImageJ analysis, as confirmed by SEM micrographs. The nanoparticle size was higher in this work than in [30], which might be attributed to changes in synthesis settings such as temperature, incubation period, plant extract type, and handling applications. Furthermore, an EDX analysis showed pure ZnO-NPs phases and a strong peak in the EDX spectrum, showing that the test sample contained pure Zinc. The EDX spectra of ZnO-NPs were obtained using a simple precipitation process using zinc as the starting material. Pure ZnO-NPs with substantial peaks have been successfully synthesized, according to the EDX spectrum. Additional peaks in the spectrum, however, were detected, suggesting that plant biomolecules were involved in nanoparticle synthesis. Throughout their examination, they found the same EDX pattern of ZnO-NPs with great purity. Reference [31] employed EDX analysis to assess the purity of ZnO-NPs and discovered pure zinc in the spectrum, as well as other peaks, suggesting that the sample was pure. XRD analysis was used to assess the size and crystallinity of the biosynthesized zinc nanoparticles. The XRD spectrum demonstrated the planar alignment and crystalline structure of ZnO-NPs. Numerous XRD reflection planes at 2 Theta and angles such as 30.73, 33.4, 35.27, 46.53, 55.2, 62.03, 67.4 degrees indicate the fcc crystal structure, as attested by JCPDS Card No. 36-1451. The average crystal size, according to Scherer’s equation (see below), is 45.32 nm. The XRD reflection planes show an fcc crystal shape with an average crystal size of 48.81 nm, which matches the International Center of Diffraction Data card (JCPDS-36-1451) and so supports the crystalline hexagonal structure synthesis [32]. UTIs are among the most prevalent bacterial illnesses, affecting around 150 million individuals worldwide each year. Despite the fact that both men and women are susceptible to UTIs, women are more likely to get infected, with up to half of all women being ill at some time in their life. Due to the increasing prevalence of multidrug-resistant (MDR) pathogens, which render antibiotic therapy for acute infection ineffective [33], current therapeutic options are inadequate. In our experiments, ZnO-NPs showed remarkable bactericidal action against UTI isolates such as *K. pneumoniae*, *S. aureus*, *E. coli*, and *P. aeruginosa*. As a result of our findings, we may assume that biosynthesized ZnO-NPs have significant bactericidal action in their natural state, and that coating pharmaceuticals can boost their effectiveness against MDR bacteria. Our findings are consistent with earlier research [34]. The antifungal activity of plant-mediated nanoparticles was also studied, with the maximum zones of inhibition (ZOI) recorded against *A. nigr*, *F. solani*, and *A. flavus* at 14.5 ± 2.5 mm, 18.2 ± 1.5 mm, and 13.8 ± 17 mm, respectively. This research supports [35]. ZnO-NPs have also been used to treat Leishmaniasis. Leishmania is a tropical and subtropical illness caused mostly by an intracellular parasite (Leishmania tropica) that is transmitted to humans by sand flies (Phlebotomus and Lutzomyia) bites [36]. Leishmaniasis is a severe worldwide health concern, according to the World Health Organization (WHO), with a wide range of clinical symptoms and possibly deadly consequences. According to one study, 1.5 to 2 million cases occur each year, placing 350 million individuals at risk [37]. At 400μg/mL, the NPs exposed to promastigote and amastigote parasite types had significant death rates of 49 ± 0.67% and 34 ± 0.93%, respectively. As a result of our research, we believe that ZnO-NPs might be used as a future treatment for cutaneous leishmaniasis. 

Cholinesterase inhibitors are among the most effective Alzheimer’s disease (AD) treatments now available, and they may be taken at any stage of the disease. A number of synthetic and natural substances have been shown to efficiently inhibit cholinesterase enzymes [29]. 

The inhibitory reaction of both enzymes is dose-dependent, according to the ZnO-NPs. The NPs were most efficient at 1000μg/mL, decreasing AChE by 55 ± 42 0.21% and BChE by 75 ± 13 0.69%.

Protein kinases are enzymes that aid cell interaction and progression through the cell cycle by allowing cells to communicate across the nuclear membrane. Protein kinases phosphorylate serine–threonine and tyrosine residues, which is significant in cancer therapy. These residues have a function in metabolism, cell apoptosis, and cellular proliferation, differentiation regulation, and control. Out-of-control phosphorylation may induce and encourage genomic alterations that can lead to cancer [38,39]. 

A dose-dependent inhibitory effect for NPs formulations was discovered. At 5 mg/mL, the largest zone of inhibition was found to be 15.2 ±.0.13, while the lowest zone of inhibition was found to be 4.3 ± 2.1 at 0.5 mg/mL. The similar sentiment is expressed in [40].

Diabetes mellitus (DM) is a term used to describe a group of metabolic illnesses characterized by persistent hyperglycemia. When insulin comes into contact with body cells, its synthesis is minimal or it is inactive, leading to a malfunction. According to the International Diabetes Federation (IDF), more than 400 million people worldwide have diabetes, with the number expected to rise to 600 million by 2045 [41]. One of the most essential tactics for mending diabetes is to reduce postprandial hyperglycemia, which may be done by blocking the two most important carbohydrate hydrolyzing enzymes in the digestive tract, α-amylase and α-glucosidase [42]. The inhibition of α-amylase and α-glucosidase was investigated at various doses of NPs ranging from 400 μg/mL to 25 μg/mL. α-amylase inhibition was found to be 47.14 ± 0.24%, and 42.69 ± 1.92% inhibition was found for α-glucosidase, with IC50s of 31 ± 2 0.67 and 35 ± 7 0.82, respectively, as previously reported by [38]. As a result, we showed that NPs may be effective in the therapy of diabetes mellitus. In all of the bioassays, ZnO-NPs displayed high antidiabetic activity. Our findings support and expand on earlier research [43]. 

One of the most critical elements in the clinical viability of nanosystems is biocompatibility. The degree of biocompatibility is determined by physical-chemical properties (such as shape, size, and surface chemistry) as well as the environment to which NPs are exposed [44]. We tested the NPs’ in vitro hemocompatibility because of this importance. Even at the highest tested dosage of 400μg/mL, the NPs displayed excellent biocompatibility against isolated human red blood cells (hRBCs). Our findings show that ZnO-NPs generated by *Monotheca buxifolia* are very biocompatible and may be employed in a variety of biological applications. Our results back up zinc nanoparticles’ bio-safety, revealing that zinc nanoparticles mediated by *Monotheca buxifolia* are stable in vivo and might be used for medicinal applications.

## 5. Conclusions

In this study, we used *Monotheca buxifolia* leaves extract as a reducing and capping agent to produce zinc oxide nanoparticles, and we investigated their numerous therapeutic and environmental implications. Proteins, carbohydrates, and phenolic compounds found in an aqueous extract of *Monotheca buxifolia* improved particle synthesis, capping, and stability. In this investigation, although *P. aeruginosa* and *A. niger* were resistant to antibiotics, they were still found susceptible to NPs formulation. Similarly, the parasite *Leishmania tropica* amastigote and promastigote forms have been demonstrated to be extremely sensitive to NPs. Significant inhibitory efficiency against α-amylase and α-glucosidase, acetylcholinesterase (AChE), and butyrylcholinesterase (BChE) was also calculated, indicating fascinating potential for the development of anti-diabetic and anti-Alzheimer’s medications. Many metallic nanoparticles are poisonous, which limits their use in clinical settings; however, biosynthesized ZnO-NPs have a high level of hemocompatibility, making them extremely compatible and non-toxic. As a result, we believe *Monotheca buxifolia* ZnO-NPs could be exploited as safe and effective nanoplatforms for a wide range of biological and environmental applications. However, the study is limited by their toxicity in in vivo animal models. We recommend that proper methodologies should be designed to check and evaluate the inherent cytotoxicity of zinc oxide nanoparticles.

## Figures and Tables

**Table 1:** Antibacterial Activity of *Monotheca buxifolia* synthesized ZnO-NPs.

Test Organisms	Activity of ZnO-NPs (mm)	Antibiotics	CLSI Standard Limit of Sensitivity (mm)	ZI of Non-Coated Antibiotics (mm)	ZI of ZnO-NPs Coated Antibiotics (mm)	Increase in the Potency of ZnO-NPs Coated Antibiotics (%)
***E. coli***	14 ± 2.63	Ciprofloxacin	21	16 ± 0.9	19.2 ± 1.5	28.4
		Imipenem	22	15 ± 1.0	18 ± 0.8	26.5
		Vancomycin	19	13 ± 1.4	14 ± 1.0	20.2
		Amoxicillin-clavulanic acid	18	10 ± 1.2	11.2 ± 1.2	22.6
***K. pneumoniae***	16 ± 2.78	Ciprofloxacin	21	15.5 ± 1.5	22.8 ± 0.4	29.0
		Imipenem	22	14 ± 0.4	21 ± 1.5	25.2
		Vancomycin	19	9 ± 1.1	12 ± 0.7	18.0
		Amoxicillin-clavulanic acid	18	6 ± 0.6	11.6 ± 1.2	22.6
***P. aeruginosa***	13 ± 2.43	Ciprofloxacin	21	12.3 ± 0.9	18 ± 1.8	27.9
		Imipenem	22	14 ± 0.6	21.3 ± 1.0	26.6
		Vancomycin	19	8 ± 1.3	10 ± 0.5	13.8
		Amoxicillin-clavulanic acid	18	10.4 ± 0.6	15.6 ± 0.9	21.4
***S. aureus***	18 ± 2.74	Ciprofloxacin	21	14.4 ± 0.9	22 ± 1.2	29.5
		Imipenem	22	16 ± 0.4	21.6 ± 0.9	22.5
		Vancomycin	19	9 ± 1.4	14.3 ± 0.7	26.9

**Table 2:** % Hemolysis of ZnO-NPs.

S.NO	Concentration (µg/mL)	% Hemolysis
1	400	1.63 ± 0.21
2	200	1.23 ± 0.35
3	100	0.82 ± 0.38
4	50	0.42 ± 0.62

## Data Availability

All required data are present in this file.
